# KERMIT: Performance indicators in electronic patient reported outcome measures: a modified Delphi

**DOI:** 10.1186/s41687-025-00898-x

**Published:** 2025-07-02

**Authors:** Nathaniel Luke Hatton, Mark Baxter, Sally Lewis, Peter S. Hall, Katie Spencer

**Affiliations:** 1 Leeds Teaching Hospital Trust, Beckett Street, Leeds, LS9 7TF England; 2 Division of Cancer Research, Ninewells School of Medicine, University of Dundee, Dundee, Scotland; 3 Welsh Value in Health Centre, Wales, UK; 4 Institute of Genetics and Cancer, University of Edinburgh, Edinburgh, Scotland; 5 Leeds Institute of Health Sciences, University of Leeds, Leeds, England

**Keywords:** ePROMS, PROMS, Electronic, KPIs, Key performance indicators, Delphi study

## Abstract

**Introduction:**

The use of electronic patient reported outcome measures (ePROMs) is increasing in routine cancer care, with benefit demonstrated in improving patient survival, satisfaction and response time. ePROMs represent a complex intervention, with successful implementation reliant upon a range of questionnaires, platform, patient and clinician characteristics alongside the wider organisational readiness and environment. Key performance indicators (KPIs) assess the performance of a system. A KPI framework would offer value in assessing ePROM implementation projects, however the outcomes and indicators of importance are not clear.

**Method:**

A modified Delphi methodology was used to define a framework of KPIs for assessing the deployment of ePROMs in routine cancer care. Potential KPIs were identified through literature searches, de-duplicated and allocated to a matrix of domains. Delphi participants were identified through a literature review and study team networks. KPIs were presented to participants for prioritisation using an online platform. A final set of KPIs was identified through two rounds of consensus with participants rating each KPI for relevance.

**Results:**

The literature search generated a list of 196 potential KPIs of which 48 were considered by 15 experts in the Delphi process. Consensus was reached to include 12 KPIs in the first round and a further 2 KPIs in the second round. Participant’s open text responses were analysed, suggesting a number of areas of debate regarding which KPIs are most pertinent.

**Discussion:**

This work provides a framework of 14 KPIs, covering those of relevance to patients, clinicians and health services and recognising the acceptability, feasibility and impact of ePROMs. This framework offers a means to appraise the implementation of ePROMs, supporting teams as they implement ePROMs in routine cancer care and other healthcare settings.

**Supplementary Information:**

The online version contains supplementary material available at 10.1186/s41687-025-00898-x.

## Background

A patient reported outcome measure (PROM) is defined as any standardised or structured questionnaire for assessing the status of a patient’s health condition, health behaviour, disability or health-related quality of life [1, 2]. PROMs allow the outcome of a clinical intervention to be measured from a patient’s perspective [1, 3]. The use of PROMs has been shown to improve health outcomes, patient experience, health services utilisation and indeed survival in patients with cancer [1]. As a consequence, the use of PROMs is rapidly increasing in routine cancer care [4].

PROM data can be used for a variety of purposes within healthcare systems. Primarily they are used to support the delivery of patient-centred clinical care [5], however, they can also provide valuable information to healthcare providers, commissioners and researchers [6]. As health services move to deliver care that is enabled by digital systems to increase efficiency and access [7], several different electronic PROM (ePROM) platforms have been developed for patients with cancer [8]. EPROMs represents a complex intervention, with many components involved in deliverly and use of ePROMs within healthcare and can be considered as events within a system [9]. Successful implementation relies upon questionnaire selection, platform and clinician characteristics alongside wider organisational readiness, environment and cost evaluations within the system [10].

Multiple ePROM platforms are already in clinical use such as MyChart and Minvera [11] Providers often conduct internal assessments of these systems, however, outcomes defining successful implementation are not clear. As a result, it is challenging to measure the success of ePROMs implementation [12]. This lack of standardised measurement means healthcare providers seeking to implement ePROMs lack information about the success or failure of different platforms to guide their decision-making. In parallel, commercial organisations seeking to develop ePROMs do not have a widely accepted framework of measures with which to assess their products and drive improvements. This is compounded by the inherent challenge of disentangling the performance of an ePROMs system from its local implementation, and thus understanding where any fault lies.

Key performance indicators (KPIs) are one way to assess the performance of a system to ensure reproducibility and reliability [6, 13]. The definition of what counts as a KPI is flexible and reflects what is considered important in an individual setting but aims to evaluate the success of a particular activity [6, 14].

In the context of ePROMs in cancer care, the definition and introduction of key performance indicators (KPIs) remain underexplored [15]. The challenge of defining KPIs arises from their dependence on the specific healthcare setting, making it difficult to standardise their application across diverse contexts.

While it is essential to have a framework of KPIs to assess the implementation of ePROMs, no such standardised framework currently exists. As a result, defining which KPIs should be used and how they should be measured remains a complex task. To overcome this, a modified Delphi approach was employed in this study [16, 17], enabling a consensus-driven process among a panel of experts to identify the most meaningful and measurable KPIs for ePROM implementation in cancer care.

## Methods

**Fig. 1:**
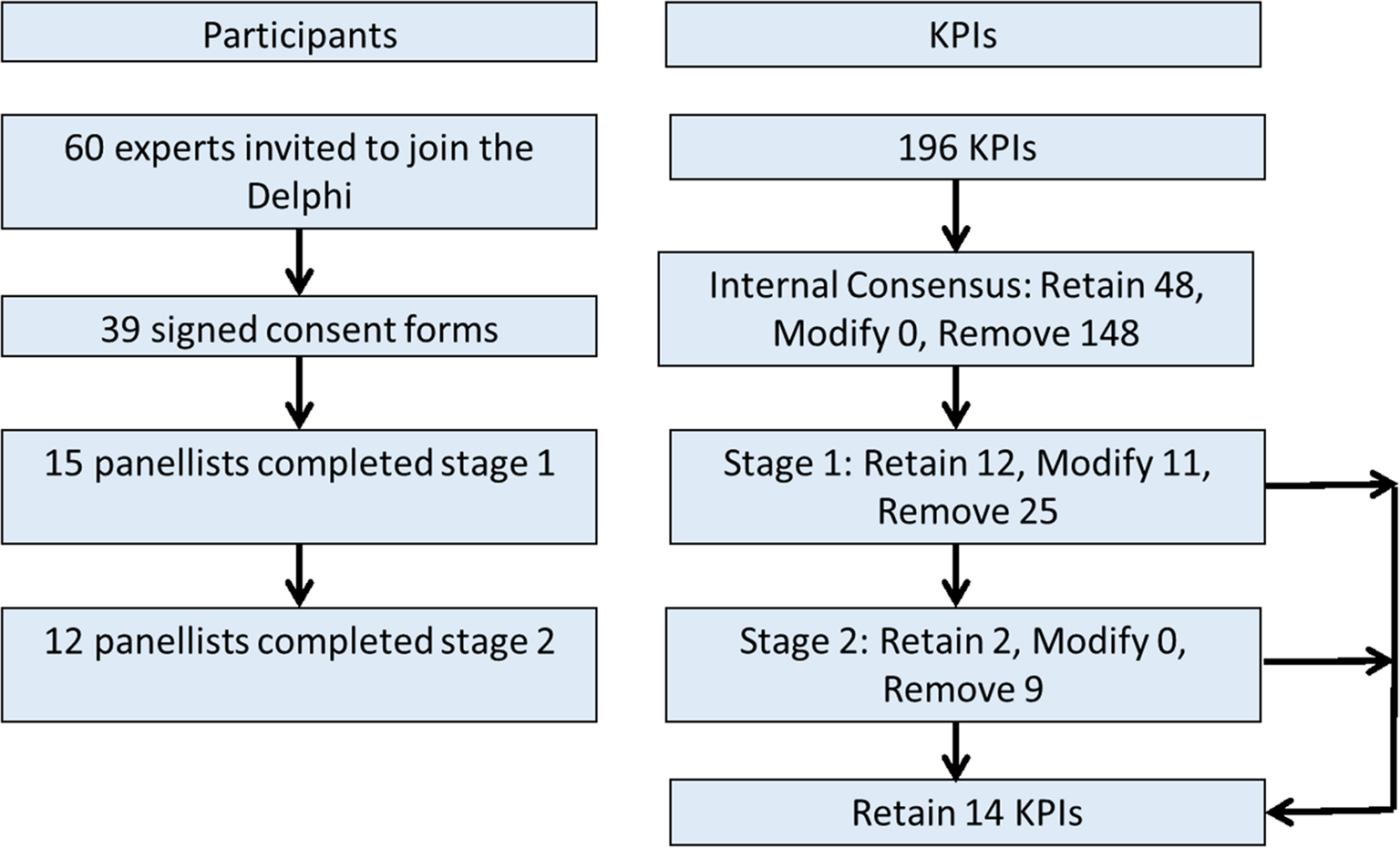
Flow diagram of study participants and questions. KPI – key performance indicator

Guided by a steering group of NLH, PH, KS, MB and SL, we conducted a multi-stage consensus building approach including: identification of potential KPIs, selection and refinement of possible KPIs and measurements; and Delphi consensus to deliver a final framework of KPIs (see Fig. 1).

### Identification of KPIs

A systematic literature search was conducted to support identification of potential KPIs for PROMs in cancer and experts in PROMs to contribute to the Delphi consensus. We searched the following databases (Medline, Embase, SCOPUS, Cinahl, Cochrane, Nice) (Supplement 1). Articles were included if they reported studies that assessed the outcomes of ePROM systems in trials and routine healthcare settings from 2000 onwards. Only studies written in English were included. Backwards and forward searching was used to identify further papers for inclusion [18, 19]. Full text review of included studies was undertaken to extract outcome measures of potential value as KPIs.

### Initial refinement of KPIs

The steering group reviewed the identified potential KPIs and removed duplicate measures and those of relevance only to a single PROM (e.g. a single symptom or questionnaire in isolation) rather than the overall system performance. This provided a selection of KPIs to be taken forward to the Delphi consensus panel. Redundant, and thus excluded, measures included those which would be infeasible to measure based either on data collected within the ePROM platform or routine clinical data available within the English National Health Service.

The authors then allocated the potential KPIs to domains based upon the stakeholder they inform (patient, professional and provider) and outcomes measured (ePROM acceptability, feasibility and impact) in order to provide structure for the planned Delphi process giving the participants a starting point to improve responses and minimising time burden for participants [20]. To minimise bias exclusion of KPIs was agreed by at least two members of the steering group.

### Participant identification

Potential participants were identified via multiple means: academic experts were identified based on the literature review; clinicians and patients were recruited through the authors contacts and sources including the Welsh Cancer Group, the Scottish Cancer PROMs Advisory Group and the National Cancer Research Institute (NCRI) living with and beyond cancer methodology subgroup; [21–23] the project was also advertised online on university websites and participants could be recruited through word of mouth [20, 24].

To minimise recruitment bias, email invitations were sent out to a broad range of participants identified in the literature. We allowed word of mouth recruitment via other participants and distribution within the networks of the study authors. There was no exclusion criteria placed on participants.

### Consensus building Delphi study

The modified Delphi process [20, 25] was conducted using REDCap, an online survey tool that has previously been used for Delphi studies [26, 27]. There are no agreed methods of how to set the sample size for Delphi surveys and there is no requirement for a statistically representative sample [28, 29].

Basic demographic information was requested from participants, including, gender, age band, country of residence, highest educational qualification, area of expertise or experience and length of experience. No identifiable participant information was collected. Participants were given the opportunity throughout the process to comment on the KPIs via free text boxes.

#### First round

All potential KPIs and an accompanying suggested measure (e.g. numerator and denominator) were presented to the Delphi panel. Participants were invited to rate the relevance of each KPI in assessing ePROM performance using a 1–7 Likert scale (seven being highly relevant and one being not relevant) [30, 31]. Participants were also invited to comment on each KPI and its proposed measurement. The comments were used to help refine KPIs and identify general themes.

“KPIs that had consensus (> 70% of participants agreeing they were strongly relevant (Likert 6 or 7) were accepted based on previous studies [17, 32, 33]. KPIs with < 50% of participants responding 6 or 7 were removed leaving those KPIs for which 50–70% reported high relevance. “Don’t know” responses were excluded from the group analysis to ensure that the reported percentage agreement and disagreement for each KPI represented the consensus among those that answered with a position.

#### Second round

KPIs with between 50 and 70% high reported relevance in round one were presented to participants again with minor modifications reflecting the first round feedback. Participants were presented with their first round comments as well as the mean and percentage agreement from the group. A simple majority was required for inclusion following the second round [34, 35], accepted and rejected KPIs from the first round were presented to the group for feedback alongside the first round feedback. The feedback was given to help people see common themes within the responses and identify trends.

The final KPI list included those accepted in the first round (based on 70% agreement) and those with majority agreement in the second round.

## Results

### Identifying KPIs and candidates

The literature search initially identified 196 potential KPIs. After the removal of duplicates and those of relevance only to a specific PROM, 48 potential KPIs were included in the first Delphi consensus round (Supplement 2), which included a potential way to measure the KPI.

### Delphi study

There were 39 participants in the first round and 15 completed responses. Basic participant demographic information for those providing completed responses is presented in Table 1. Data from incomplete responses was not included due to it’s limited nature.

**Table 1:** Demographic information of Delphi participants. ePROMS – electronic patient reported outcome measure

**Age Group**	
31–35	2
36–40	2
41–45	3
46–50	3
> 51	5
**Median age group**	41–50
**Sex**	
Female	60%
Male	40%
**Location**	
United Kingdom	8
Europe	2
Rest of world	5
**Educational level**	
PhD degree	12
Master’s degree	3
**Current work**	
ePROMs	8
researchers Patient	1
Academic researchers	4
Organisation	2
**Length of work experience**	
0–5	5
6–10	4
11–15	3
> 16 years	3

In the first round, consensus was achieved on the inclusion of 12 KPIs, with 25 rejected and 11 being undecided.

All participants were invited to the second round of which 12 participated. For the 11 undecided KPIs, the participants were asked if the KPIs are relevant or not relevant. Two KPIs were relevant based on the previous criteria and 9 were not relevant.

A total of 14 KPIs were included in the final ePROM KPI framework (see Table 2).

### Participant comments

Participants provided written feedback during each Delphi round. These comments were analysed for general trends and themes. These themes are presented below in italics.

#### KPIs could be difficult to measure and define

Several challenges defining and measuring KPIs were reported. For example, KPIs can be defined in different ways and their acceptability changes depending on the definition. Even if the KPI can be measured and defined well, it may need different types of measurement throughout the life cycle of the system. For example, the acceptability of an ePROMs system may need different types of measurements for patients and health professionals.

#### Interactions of the KPI within the wider system

Multiple participants fed back concerns about the extent to which potential KPIs were able to specifically assess the ePROM implementation under review as opposed to assessing the ePROM *and* wider health system in combination (the two being interconnected, reflecting how the ePROM system is embedded in clinical practice). Disentangling these was felt to be challenging but important to provide an assessment of the ePROM system independently of any challenges faced by the wider health service.

Accordingly, while good outcomes suggest that the ePROM system may perform well, good clinical care may mask an underperforming system, whilst conversely an underperforming outcome does not necessarily imply that the ePROM system is performing poorly.

Further, participants identified that a number of the excluded KPIs did not measure the ePROM system but the PROM questionnaire tool itself. It was also felt that some measures were more suited to research settings and had less clinical relevance.

#### Measurement ignoring clinical burden

Some of the feedback reflected concern about clinical workloads as some KPIs required clinician feedback which would add to workload and not be used in routine patient care because of a lack of take up.

**Table 2:** KPI framework structured by patient, professional and provider

	Patient	Professional	Provider
**Acceptability**	**1 Acceptability of ePROMs**: Proportion of patients who felt that the completion of ePROMs was a good use of their time **2 Quality of life**: Proportion of patients who feel that the ePROMs system measures the things that impact on their Quality of life	**1 Healthcare professional’s satisfaction with service**: Proportion of healthcare professionals satisfied with ePROMs contribution to service	**1 Privacy**: Proportion of patients who feel their data is safe
**Feasibility**	**1 Interpretability of system**: Proportion of system users who find the system easy to use and understand	**1 Communication**: Proportion of health professionals who feel the ePROMs system communicates and integrates effectively with other programmes and software **2 Clinic flow integration**: Proportion of Health professionals who report the ePROMs system is integrated into clinic flow	**1 Missing data**: Proportion of missing items per questionnaire (measured over a specified time period **2 Utilisation**: Percentage of users and healthcare professionals using ePROMs system over a specified time period.
**Impact**	**1 Symptoms monitoring**: Proportion who feel the ePROMs helps with timely symptom recognition **2 Shared decision making**: Proportion of patients who report being asked their goals and preferences of care **3 Improves patient clinician communication**: Proportion of patients who feel using the ePROMs system improves communication with their clinical team of symptoms	**1 Symptom recognition**: Proportion of healthcare professionals who feel ePROMs improves symptom recognition	**1 Unscheduled contact**: Proportion of unscheduled patient initiated contact per month

## Discussion

The KERMIT project is the first reported attempt to create a framework of KPIs for use when assessing the implementation of ePROMs in routine cancer care using a modified Delphi Study. ePROMs are being implemented and increasingly used in routine cancer care with trials showing multiple benefits to their use in clinical practice [1]. However routine ePROM implementation remains challenging in the real-world setting, because of cost, clinical factors and organisational factors such as readiness and environment [1, 36]. To support the appraisal of ePROM implementation projects, we conducted a Modified Delphi process to establish a framework of KPIs. The framework we present is relevant to routine clinical settings and the included KPIs are readily collected in this setting.

Most ongoing research relating to ePROMs focuses on specific ePROMs systems, showing benefit in specific cancer sites [37] or looking at how to improve the specific ePROMs system within practice [38]. Fewer studies are looking at the fundamentals to success of the system within the wider health system and how success should be measured [38, 39].

Industry and health systems will be increasingly faced with a diverse market with a range of similar products. This creates difficulty in selecting a system, and when implementing the selected system, a challenge in how to measure its success [36]. Our framework helps establish a range of measures that can be used to assess this implementation.

This project has several strengths supporting the robust development of a framework of KPIs to assess ePROM implementation with adaptive feedback leading to addition KPIs. The literature search was developed to be inclusive in order to find potential KPIs that have been used within a research setting, alongside implementation studies. This was done to help give a broad range of potential KPIs that have been used in other settings and could be relevant to our study. Further, we obtained a variety of perspectives from different stakeholder groups, in different countries, as well as having a good response rate. This helps to reflect a range of viewpoints and ensures results are relevant and applicable, as has been shown to be important in previous Delphi studies [18].

There are, however, limitations of this project, despite the rigorous literature review and inclusive approach to participant recruitment, our relatively small sample size of Delphi participants could be considered a limitation. While this is consistent with the Delphi methodology which does not require a statistically representative sample, it may limit the breadth of perspective and diversity of opinions represented. We did take steps to minimise bias such as open recruitment and inviting a diverse group of experts to participate. Participation was voluntary and thus some participants might not of chosen to take part given a lack of time.

There was also relatively less representation of clinical perspectives (e.g. clinicians, operational management and patients) than academics. This could mean those views were not expressed as strongly as others in the Delphi process [40]. We attempted to balance this by providing a range of KPIs at the start of the Delphi processes that reflects the literature at the time of the survey. We also used the individual feedback from the group to help structure the survey and responses, giving consideration to the different viewpoints. Finally, we gave practical examples of how the KPI might be relevant to clinical practice with a suggested measure (Supplement 2). Additionally, the qualitative nature of the study means that results are based on expert opinions and consensus rather than empirical evidence from real world implementation. The abstract nature of some KPIs also means that their applicability and effectiveness in real world settings remain uncertain. While the study focused on defining KPIs we did not explore in depth how these indicators would be practically integrated into clinical workflows. The real world feasibility and impact of implementing these KPIs remain uncertain and require further evaluation.

There could be a concern that in real world implementation some of the KPIs are not practical in clinical practice or indeed are difficult to measure depending on clinical setting, resource and wider digital set up. Challenges of measurement may be overcome by keeping the KPI but finding a different, more practical way to measure it within a given clinical setting. An example of this could be giving certain KPIs more weighting then others in specific circumstances, this however was out of scope of this specific study. The need for and success of this will only become apparent through testing in a real-world setting.

The findings of this study could be relevant to ePROMs implementation with other disease sites. This study looked specifically at cancer. Most of the KPIs would be relevant to other disease sites, however there might be specific ones for other diseases which would require further evaluation which is outside the scope of this study.

At a practical level there is also a balance to be struck between the benefits and burdens of data collection. The presented framework minimises clinician workload from measurement through inclusion of only four KPIs requiring clinician input. These could be asked via the ePROM platform as occasional pop-ups but the burden on clinicians must still be recognised and the risk that clinicians do not complete these as they interrupt clinical workflows [41]. The balance between burden and benefit is likely to vary with clinical setting and was considered to be outside the scope of this study.

In summary, this Delphi study highlights the difficulties disentangling measures of the health system from the actual ePROM system and its implementation. ePROM systems are created by a range of developers from academic, clinical or commercial backgrounds and the rationale for development and indeed procurement may also vary. Similarly, clinicians and other health professionals may use ePROMs in different ways and as such ePROMs systems may be used for reasons other than their intended purpose. ePROMs implementation relies upon the wider system as a perfect ePROM measurement might not work if the wider system it is implemented in is not able to support it. For example, missing data could be due to limitations in the ePROM system or the wider environment, potentially reflecting reduced patient engagement if clinical teams do not consider ePROMs in clinical consultations [36]. KPIs should therefore be interpreted in the context of the clinical environment.

This study, following a Delphi consensus process creates a framework of KPIs which can be used to assess ePROMs implementation. Further work is required to use this framework in real world settings and validate its implementation.

## Electronic supplementary material

Below is the link to the electronic supplementary material.

Supplementary Material 1

Supplementary Material 2

## Data Availability

All material will be made available upon reasonable request.
