# Supplementary material for: KERMIT: Performance indicators in electronic patient reported outcome measures: a modified Delphi
Source: J Patient Rep Outcomes. 2025 Jul 2;9:81. doi: 10.1186/s41687-025-00898-x (PMC12222585; doi:10.1186/s41687-025-00898-x)
Supplement: Supplementary file 1 — Supplementary Material 1 [file 41687_2025_898_MOESM1_ESM.docx]

**Ovid MEDLINE(R) ALL <1946 to May 21, 2021> 295**

1 exp Quality Indicators, Health Care/ 22724

2 (Performance adj2 (indicator* or measure* or Improvement* or Metric*)).tw,kw. 40116

3 (quality adj2 (indicator* or measure* or improvement* or Metric* or assessment*)).tw,kw. 104106

4 "Quality of Health Care"/ 74717

5 exp Quality Improvement/ 28564

6 "Outcome and Process Assessment, Health Care"/ or Outcome Assessment, Health Care/ 103935

7 quality assurance, health care/ or total quality management/ 68003

8 (KPI* or KPM*).tw,kw. 1238

9 Performance evaluation*.tw,kw. 7114

10 (Outcome* adj2 (measure* or Indicator* or assessment*)).tw,kw. 290130

11 1 or 2 or 3 or 4 or 5 or 6 or 7 or 8 or 9 or 10 642695

12 exp Patient Reported Outcome Measures/ 8361

13 (patient* report* adj2 (outcome* or measure*)).tw,kw. 23645

14 (PROM or PROMS).tw,kw. 5062

15 (ePROM or ePROMS or e-PROM or e-PROMS or epro or e-Pro).tw,kw. 257

16 Self Report/ 36287

17 self-report*.tw,kw. 169583

18 12 or 13 or 14 or 15 or 16 or 17 206674

19 exp Neoplasms/ 3469292

20 (cancer* or neoplasm* or malignan* or oncology or tumo?r* or sarcoma* or carcinoma* or leuk?emia* or lymphoma* or mesothelioma* or pseudomyxoma* or glioblastoma*).tw,kw. 3757350

21 Medical Oncology/ or Radiation Oncology/ or Integrative Oncology/ or Psycho-Oncology/ or Oncology Nursing/ or Surgical Oncology/ 32486

22 19 or 20 or 21 4602104

23 exp Electronics/ 38370

24 exp Medical Records Systems, Computerized/ or exp Electronic Health Records/ or Information systems/ 60979

25 exp Telemedicine/ 34552

26 Software/ 114192

27 Digital.tw,kw. 136445

28 Electronic.tw,kw. 267952

29 Electronic Medical Records.mp. 11229

30 eHeatlh.mp. 0

31 telemedicine.mp. 35648

32 23 or 24 or 25 or 26 or 27 or 28 or 29 or 30 or 31 605667

33 11 and 18 and 22 and 32 295

**Notes on OVID Medline searching**

General

- Used tw.kw to be more specific in finding articles from the title of abstract rather than searching the whole text
- EXP means it selects all articles which have also been tagged with any more specific narrower terms in the thesaurus. The decision to EXP or limit was based on the number of hits that came back on a case by case bases.

For Each Search code above.

1. Identifying the KPIs is the most important aspect so that comes first, Exp means it selects all articles which have also been tagged with any more specific narrower terms in the thesaurus
2. Performance – no difference in spelling English/American adj2 means will search for Word x x indicator* either side
3. Quality – no difference in spelling English/American adj2 means will search for Word x x indicator* either side
4. Quality of health care, if exploded several million articles, thus kept as subject heading without exploding
5. Exp Quality Improvement it selects all articles which have also been tagged with any more specific narrower terms in the thesaurus
6. Subject heading search of outcome and process or outcome assessment
7. Subject heading search of quality assurance and total quality management
8. Search for KPI* or KPM* as not going to confuse with other words.
9. Performance evaluation no change of English vs American
10. Outcome no difference in spelling English/American adj2 means will search for Word x x indicator* either side
11. Combination of the first searching for KPIs with Or
12. Explode subject heading Patient reported outcome measures
13. Search for using adj patient reported outcomes
14. Search for the acroymns using specifics as * will cause other words to be found.
15. Searching for ePROMs as a separate term
16. Subject heading self reports as might include PROs
17. Search for self reports as might include Pros
18. Combination excluding self reports
19. Explode subject heading of neoplasm
20. Cancer search term changed to tw,kw to search abstract and title
21. Search for Oncology subject terms
22. Combination of cancer search terms
23. Subject heading Electronic
24. Explode of subject terms relate to Electronic and health records
25. Exp of subject telemedicine as could contain electronic PROMS systems
26. Software not exploded but could contain electronic proms systems
27. Digital search in title and abstract as could be included in name
28. Search electronic as could be incluced in title or abstract of EPROMs systems
29. Electronic medical records searched for phrase as unlikely to be included in title of abstract but could in body of text
30. eHealth search in text as unlikely to be found in abstract or title
31. telemedicine searched in text as could be found outside of abstract or title

**Embase Classic+Embase <1947 to 2021 May 24>**

1 exp health care quality/ 3464781

2 (Performance adj2 (indicator* or measure* or Improvement* or Metric*)).tw,kw. 53003

3 (quality adj2 (indicator* or measure* or improvement* or Metric* or assessment*)).tw,kw. 162449

4 exp total quality management/ 72189

5 exp outcome assessment/ 603349

6 exp quality control/ 435024

7 (KPI* or KPM*).tw,kw. 2074

8 Performance evaluation*.tw,kw. 9587

9 (Outcome adj2 (measure* or Indicator* or assessment*)).tw,kw. 339291

10 1 or 2 or 3 or 4 or 5 or 6 or 7 or 8 or 9 4042813

11 exp patient-reported outcome/ 31102

12 (patient* report* adj2 (outcome* or measure* or assessment*)).tw,kw. 44768

13 (PROM or PROMS).tw,kw. 8643

14 (ePROM or ePROMS or e-PROM or e-PROMS or epro or e-Pro*).tw,kw. 8787

15 exp self report/ 129506

16 self-report*.tw,kw. 223923

17 11 or 12 or 13 or 14 or 15 or 16 314806

18 exp malignant Neoplasms/ 3475281

19 exp cancer patient/ 508688

20 (cancer* or neoplasm* or malignan* or oncology or tumo?r* or sarcoma* or carcinoma* or leuk?emia* or lymphoma* or mesothelioma* or pseudomyxoma* or glioblastoma*).tw,kw. 5346280

21 Oncology/ 155094

22 18 or 19 or 20 or 21 5839201

23 exp medical Electronics/ 363

24 exp electronic health record/ or exp electronic medical record/ 83929

25 exp Information Systems/ 168161

26 exp Telemedicine/ or exp telehealth/ 58389

27 exp healthcare software/ 309

28 Digital.tw,kw. 184789

29 Electronic.tw,kw. 333365

30 Electronic Medical Records.tw,kw. 22181

31 eHeatlh.tw,kw. 2

32 telemedicine.tw,kw. 22000

33 23 or 24 or 25 or 26 or 27 or 28 or 29 or 30 or 31 or 32 726792

34 10 and 17 and 22 and 33 1909

**SCOPUS 84**

**KPIs**

(Quality w/2 (Assessment* OR Indicator* Or Improvement*)) OR ( performance W/2 ( indicator* OR improvement* OR metric* OR evaluation) ) OR "quality of health care" OR ( outcome W/2 ( measure* OR indicator* OR Assessment*) ) OR kpi* OR kpm* )

**PROMS**

"PROM" or "PROMs" or "ePROM" or "ePROMs" or “ePRO*” or “e-PRO*” or "self report" or ("patient* report*" w/2 ("outcome*" or "measure*"))

**Cancer**

Cancer* or Neoplasm* or Malignan*

**Electronic**

“Electronic” or “digital” or “online” or “Healthcare Software” or “Telemedic*” or “eHealth”

**Search for SCOPUS (NK comments listed above)**

( TITLE-ABS-KEY ( ( quality W/2 ( assessment* OR indicator* OR improvement* ) ) OR ( performance W/2 ( indicator* OR improvement* OR metric* OR evaluation* ) ) OR "quality of health care" OR ( outcome W/2 ( measure* OR indicator* OR assessment* ) ) OR kpi* OR kpm* ) AND TITLE-ABS-KEY ( "PROM" OR "PROMs" OR "ePROM" OR "ePROMs" OR “epro*” OR “e-pro*” OR "self report" OR ( "patient* report*" W/2 ( "outcome*" OR "measure*" ) ) ) AND TITLE-ABS-KEY ( cancer* OR neoplasm* OR malignan* ) AND TITLE-ABS-KEY ( “electronic” OR “digital” OR “online” OR “healthcare AND software” OR “telemedic*” OR “ehealth” ) )

**Results 84**

**Notes for SCOPUS**

- Search broken down initially into the 4 domains/themes and built together using the OR function before combining the domains with AND function.
- Mixture of Key word searching in Title, Abstract and Keywords and the MeSH searching of linked tree areas.

**Data search of CINAHL 170**

S22 S8 AND S13 AND S17 AND S21 170

S21 S18 OR S19 OR S20 207,321

S20 TI ( “online” or “Healthcare Software” or “Telemedic*” or “eHealth” ) OR AB ( “online” or “Healthcare Software” or “Telemedic*” or “eHealth” ) 83,327

S19 (MH "Digital Technology+") OR TI "Digital" OR AB "Digital 41,043

S18 (MH "Electronic Health Records+") OR TI "Electronic" OR AB "Electronic" ) 92,516

S17 S14 OR S15 OR S16 760,972

S16 TI "malignan*" OR AB "malignan*" 74,737

S15 (MH "Cancer Patients") OR TI (Cancer* or oncology or tumo?r* or sarcoma* or carcinoma* or leuk?emia* or lymphoma* or mesothelioma* or pseudomyxoma* or glioblastoma*) OR AB (Cancer* or oncology or tumo?r* or sarcoma* or carcinoma* or leuk?emia* or lymphoma* or mesothelioma* or pseudomyxoma* or glioblastoma*) 538,214

S14 (MH "Neoplasms+") OR TI "Neoplasm*" OR AB "Neoplasm*" 577,879

S13 S9 OR S10 OR S11 OR S12 112,659

S12 (MH "Self Report") or (MH "Self Assessment") OR TI "self report" OR AB "self report" 97,906

S11 TI ( PROM Or PROMs or ePROM or ePROMs or ePRO ) OR AB ( PROM Or PROMs or ePROM or ePROMs or ePRO ) 2,117

S10 TI ( (patient reported N2 (Outcome* or Measure* or assessment* or indicator*)) ) OR AB ( (patient reported N2 (Outcome* or Measure* or assessment* or indicator*)) ) 14,196

S9 (MH "Patient-Reported Outcomes+") 3,329

S8 S1 OR S2 OR S3 OR S4 OR S5 OR S6 OR S7 838,745

S7 (MH "Outcomes (Health Care)") OR AB "outcomes health care" or TI "outcomes health care" 54,327

S6 TI ( KPI* or KPM* ) OR AB ( KPI* or KPM* ) OR TI key performance indicators OR AB key 667

S5 (MH "Quality Improvement+") OR TI "quality improvement" ) OR AB "quality improvement" ) 78,915

S4 TI ( Performance w2 (Indicator* or Measure* Or Improvement* or Metric* or evaluation*) ) OR AB ( Performance w2 (Indicator* or Measure* Or Improvement* or Metric* or evaluation*) ) 16,383

S3 TI ( Performance w2 (Indicator* or Measure* Or Improvement* or Metric* or evaluation*) ) OR AB ( Performance w2 (Indicator* or Measure* Or Improvement* or Metric* or evaluation*) ) 15,031

S2 (MH "Clinical Indicators") 12,725

S1 (MH "Quality of Health Care+") OR (MH "Quality Assessment+") OR (MH "Quality Improvement+") OR TI "Quality of health care" OR AB "Quality of health care" 821,147

**Cochrane – 185**

Further selected to include just cancer

#1 MeSH descriptor: [Quality Indicators, Health Care] explode all trees 554

#2 MeSH descriptor: [Quality of Health Care] explode all trees 460745

#3 ("Performance Indicator*" OR "Performance Measure*" OR "Performance Improvement" Or "Performance Metric*"):ti,ab,kw 953

#4 ("KPI*" OR "KPM*"):ti,ab,kw 119

#5 ("Quality Indicator*" OR "Quality Measure*" or "Quality Metric*" or “quality Improvement” Or “quality assessment”):ti,ab,kw 4061

#6 "Quality of Health Care":ti,ab,kw 1027

#7 MeSH descriptor: [Quality Improvement] this term only 743

#8 "Performance Evaluation":ti,ab,kw 197

#9 ("Outcome Measure*" or "Outcome Indicator*” or “Outcome Metric*"):ti,ab,kw 24571

#10 MeSH descriptor: [Quality Assurance, Health Care] explode all trees 3291

#11 #1 or #2 or #3 or #4 or #5 or #6 or #7 or #8 or #9 or #10 477916

#12 MeSH descriptor: [Patient Reported Outcome Measures] explode all trees 706

#13 ("PROM" or "PROMs" or "ePROMs" or "ePROM" or “ePRO”):ti,ab,kw 1273

#14 (patient near/2 (outcome* or measure*)):ti,ab,kw 32004

#15 MeSH descriptor: [Self Report] explode all trees 2366

#16 "Self Report*":ti,ab,kw 16363

#17 #12 or #13 or #14 #15 or #16 18225

#18 MeSH descriptor: [Neoplasms] explode all trees 81536

#19 (cancer* or neoplasm* or malignan* or oncology or tumo?r* or sarcoma* or carcinoma* or leuk?emia* or lymphoma* or mesothelioma* or pseudomyxoma* or glioblastoma*):ti,ab,kw 236670

#20 #18 or #19 241610

#21 MeSH descriptor: [Medical Records Systems, Computerized] explode all trees 631

#22 MeSH descriptor: [Electronic Health Records] explode all trees 379

#23 MeSH descriptor: [Electronics] explode all trees 843

#24 MeSH descriptor: [Information Systems] this term only 59

#25 MeSH descriptor: [Telemedicine] explode all trees 2764

#26 MeSH descriptor: [Software] this term only 953

#27 ("Electronic" or "Digital" or "Electronic Medical Records" or "eHeatlh"):ti,ab,kw 28168

#28 #21 or #22 or # 23 or #24 or #25 or #26 or #27 194113

#29 #11 and #17 and #20 and #28 185
