# Supplementary material for: KERMIT: Performance indicators in electronic patient reported outcome measures: a modified Delphi
Source: J Patient Rep Outcomes. 2025 Jul 2;9:81. doi: 10.1186/s41687-025-00898-x (PMC12222585; doi:10.1186/s41687-025-00898-x)
Supplement: Supplementary file 2 — Supplementary Material 2 [file 41687_2025_898_MOESM2_ESM.docx]

|  | **Patient** | **Professional** | **Provider** |
| --- | --- | --- | --- |
| **Acceptability** | **1 Patient satisfaction with service:** Patient satisfaction with ePROMs using a rateability scale **2 Acceptability of ePROMs:** Proportion of patients who felt that the completion of ePROMs was a good use of their time **3 Improves patient clinician communication:** Proportion of patients who feel using the ePROMs system improves communication with their clinical team of symptoms  **4 Quality of life:** Proportion of patients who feel that the ePROMs system measures the things that impact on their Quality of life | **1 Healthcare professional's satisfaction with service:**  Proportion of healthcare professionals satisfied with ePROMs contribution to service **2 Healthcare professional's satisfaction with care:**  Proportion of healthcare professionals satisfied with ePROMs contribution to care **3 Communication between professionals:**  Proportion of health professionals who feel ePROMs improves communication between healthcare professionals | **1 Privacy:**  Proportion of users who feel the data is securely maintained **2 Cost of maintenance:**  Health managers on a ratability scale feel the cost of maintenance is acceptable for their organisation  **3 Internet:**  Proportion of users who feel they have adequate access to the internet **4 Clinic flow integration:**  Proportion of Health professionals who report the ePROMs system is integrated into clinic flow |
| **Feasibility** | **1 Interpretability of system:** Proportion of system users who find the system easy to use and understand **2 Patient education:** Proportion of Patients who feel the their knowledge was helped with the ePROMs system | **1 Time spent training in system:**  Was the time spent learning to use the ePROMs system too much, too little or just right **2 Healthcare workload:**  Proportion of healthcare professionals who feel system increases workload **3 Severity alerts:** Number of symptom severity alerts per patient per month that have to be actioned by health professional | **1 Missing data:**  Proportion of missing items per questionnaire (measured over a specified time period **2 Completion of questionnaire:**  Proportion of available questionnaires attempted **3 Utilisation:**  Percentage of users and healthcare professionals using ePROMs system over a specified time period **4 System implementation:**  Proportion of health professionals, who rate system implementation highly on a rateability scale per month **5 Ability to Implement:**  Extent to which implementation team feel that sufficient technical capability (software, hardware and technical expertise) was available to implement ePROM system, on a rateability scale. **6 Communication:**  Proportion of health professionals who feel the ePROMs system communicates and integrates effectively with other programmes and software  **7 Clinic flow integration:**  Proportion of Health professionals who report the ePROMs system is integrated into clinic flow |
| **Impact** | **1 Timeliness of care:**  Proportion of patients who feel the timeliness of symptom recognition was improved by the ePROMs system **2 Compliance with medications:**  Proportion of prescribed medication doses recorded as taken by the patient **3 Number of medications changed in response to ePROMs response:**  Proportion of patients using as required medications, measured against symptom burden  **4 Quality of Care:**  Proportion of patient who feel ePROMs improves quality of care **5 Symptoms monitoring:**  Proportion who feel the ePROMs accurately reflects changes of my symptoms **6 Reflects symptoms:**  Proportion who feel ePROMs captures all the symptoms they are feeling **7 Overall distress:**  Proportion of patients on a rateability scale rate ePROMs captures my overall distress  **8 Coping:**  Proportion of patients who feel it measures their feeling of coping **9 Engagement self care:**  Proportion of patients who report ePROMs increases independence in symptom management **10 Shared decision making:**  Proportion of patients who report being asked their goals and preferences of care | **1 Length of clinical encounter:**  Proportion of health professionals who feel that length of clinical encounter is not extended by ePROMs **2 Symptom recognition:**  Proportion of healthcare professionals who feel ePROMs improves symptom recognition **3 Effect on clinical decision making:**  Proportion of health professionals who feel ePROMs helps support clinical decisions **4 Consideration of patient preference and needs:** Proportion of healthcare professionals who feel ePROMs help ensure patient preferences and needs are recognised **5 Response to treatment:**  The proportion of patients were response to treatment is measured? **6 Overall survival:**  Overall survival of patients using ePROMs  **7 Quality adjusted survival:**  Quality adjusted survival **8 Physician initiated contact:**  Proportion of patients contacted by physician because of ePROMs system per month **9 Care is coordinated:**  Proportion of health professionals who feel ePROMs improves communication between healthcare professionals | **1 Patterns of use:**  Proportion of users logging in per month **2 Attendance at the accident and emergency room:**  The proportion of patients attending accident and emergency per month **3 Length of hospital stay:**  The length of hospital stay for patients per month **4 Hospitalisation:**  Proportion of patients admitted to hospital per month **5 Unscheduled contact:**  Proportion of unscheduled patient initiated contact per month **6 Value for money:** Health managers who on a rateability scale feel the financial costs of ePROMs and implementation are affordable for the organisation |

|  | **Patient** | **Professional** | **Provider** |
| --- | --- | --- | --- |
| **Acceptability** | 1 Patient satisfaction with service 2 Acceptability of ePROMs  3 Improves patient clinician communication  4 Quality of life | 1 Healthcare professional's satisfaction with service 2 Healthcare professional's satisfaction with care 3 Communication between professionals | 1 Privacy 2 Cost of maintenance 3 Internet 4 Clinic flow integration |
| **Feasibility** | 1 Interpretability of system 2 Patient education | 1 Time spent training in system 2 Healthcare workload 3 Severity alerts | 1 Missing data 2 Completion of questionnaire  3 Utilisation 4 System implementation 5 Ability to Implement 6 Communication 7 Clinic flow integration |
| **Impact** | 1 Timeliness of care 2 Compliance with medications 3 Number of medications changed in response to ePROMs response 4 Quality of Care 5 Symptoms monitoring 6 Reflects symptoms 7 Overall distress 8 Coping 9 Engagement self care 10 Shared decision making | 1 Length of clinical encounter 2 Symptom recognition 3 Effect on clinical decision making 4 Consideration of patient preference and needs 5 Response to treatment 6 Overall survival 7 Quality adjusted survival 8 Physician initiated contact 9 Care is coordinated | 1 Patterns of use 2 Attendance at the accident and emergency room 3 Length of hospital stay 4 Hospitalisation 5 Unscheduled contact 6 Value for money |
